# A Scalable Sub-Picosecond TDC Based on Analog Sampling of Dual-Phase Signals from a Free-Running Oscillator

**DOI:** 10.3390/s25175577

**Published:** 2025-09-06

**Authors:** Roberto Cardella, Luca Iodice, Lorenzo Paolozzi, Thanushan Kugathasan, Antonio Picardi, Carlo Alberto Fenoglio, Pierpaolo Valerio, Fulvio Martinelli, Roberto Cardarelli, Giuseppe Iacobucci

**Affiliations:** 1 Department of Nuclear and Particle Physics (DPNC), University of Geneva, 24 Rue du Général-Dufour, 1211 Geneva, Switzerland; luca.iodice@unige.ch (L.I.); lorenzo.paolozzi@unige.ch (L.P.); thanushan.kugathasan@unige.ch (T.K.); carlo.fenoglio@unige.ch (C.A.F.); giuseppe.iacobucci@unige.ch (G.I.); 2 EM Microelectronic—Marin SA, Sors 3, 2074 Marin, Switzerland; 3 INFN Sezione di Roma Tor Vergata, Via della Ricerca Scientifica, 00133 Roma, Italy

**Keywords:** sub-picosecond, time-to-digital converter, TDC, free-running ring oscillator, timing

## Abstract

This work presents a novel time-to-digital converter based on the analog sampling of dual-phase periodic signals generated from a free-running oscillator. A proof-of-concept ASIC, implemented in 130 nm CMOS technology, achieves an average single-shot precision of 0.9 ps-rms for time intervals up to 3 ns, with a best performance of 0.79 ps-rms. It maintains a precision below 3.7 ps-rms for intervals up to 25 ns. The design demonstrates excellent linearity, with a peak-to-peak differential nonlinearity of 0.56 LSB and a peak-to-peak integral nonlinearity of 1.43 LSB. The free-running oscillator is shareable across multiple channels, enabling power consumption of approximately 4.1 mW per channel and efficient area utilization. These features make the design highly suitable for detection systems requiring picosecond-level precision and high channel density, such as silicon pixel sensors, SPADs, LiDARs, and time-correlated single-photon counting systems. Furthermore, the architecture shows strong potential for use in high-count-rate applications, reaching up to 22 Mcps.

## 1. Introduction

Time-to-Digital Converters (TDC) with measurement precisions of a few picoseconds have attracted significant interest for several applications, such as Light Detection And Ranging (LiDAR) systems, medical imaging, life sciences, time-correlated single-photon counting (TCSPC), quantum detection, spectroscopy, and high-energy physics (HEP) [1,2,3,4,5]. The need to integrate hundreds of measurement channels into large systems, as in SPADs large matrices, requires low power consumption on the order of few mW per channel, small area, and scalability. Another relevant parameter is the count-rate (measurement rate), defined as the number of counts per second (cps) that can be sustained by the system. A full-scale range (FSR) of several tens of nanoseconds at event rates up to 100 Mcps is particularly relevant for time-correlated single-photon counting (TCSPC) in life-science applications and for high-energy physics experiments such as the CERN LHC, where bunch collisions occur at a 40 MHz rate.

Several TDC architectures and implementations are available in the literature, but none of them succeed in satisfying all these requirements. Recent findings demonstrated the feasibility of picosecond-level time resolutions on discrete components or FPGAs [6]. This approach allows for fast development, but it is limited to a few measurement channels, and it is not applicable to high-channel-density systems. This requirement could be fulfilled only by the implementation of an Application-Specific Integrated Circuit (ASIC). The state of the art in integrated TDC precision targets Phase-Locked Loop (PLL) architectures, where the FSR is limited to 1 ns [7,8]. Developments targeting photon-counting systems demonstrate picosecond-scale time precision but struggle to satisfy the low-power and small-area requirements of high-channel-density systems [9].

The novel TDC architecture proposed in this work is based on a Time-to-Analog Converter (TAC) that samples dual-phase signals generated by a free-running oscillator. A proof-of-concept ASIC implemented in a commercial 130 nm CMOS technology demonstrates the feasibility of this approach, and detailed measurement results are presented to validate its performance. The paper is structured as follows: Section 1 presents a brief summary of the most common TDC architectures; Section 2.1 describes the operating principle behind the proposed architecture; Section 2.2 describes the proof-of-concept ASIC design; Section 2.3 shows the analysis of the single-shot precision and the source of jitter in this architecture; and Section 2.4 presents the measurement setup used in this work. Finally, the results are presented and discussed in Section 3 and conclusions are drawn in Section 4.

### State-of-the-Art TDCs

The main metrics used to describe a TDC are as follows:

- *latency*, which corresponds to the time between the end of the measured time interval and the availability of the measurement result; architectures with zero latency are usually referred to as flash TDCs;
- *dead time*, which refers to the period during which a measurement channel is unavailable after the end of the measured time interval;
- *maximum count rate*, which is limited by the dead time and defines the highest sustainable event rate for the system;
- *resolution*, defined as the minimum resolvable time interval;
- *single-shot precision*, or simply precision, which refers to the agreement between repeated measurements, often described as the root mean square (RMS) deviation;
- *full-scale range*, corresponding to the maximum time interval that the TDC can measure;
- *average power consumption* required per channel.

We report here a summary of the main architectures that can be found in the literature in relation to the scope of this paper. We invite the reader to see recent review papers for a broader view [1,6]. Most of the architectures can be grouped into three main families: delay-line TDC, Vernier TDC and TAC-based TDC. Delay-line flash TDCs are based on a single reference-clock delay chain, which is composed of several buffer stages. The output of each buffer is sampled at the arrival of an event. The time interval between the injection of the reference clock in the delay line and the arrival of the event is therefore encoded in the sampled outputs. While this architecture is suitable for high event rates due to its zero dead time and latency, as well as its relatively low power and complexity, it imposes a technology-dependent resolution limit, since the resolution corresponds to the minimum delay of a single buffer stage. The precision worsens over longer time intervals due to process variations and mismatch between delay elements [10]. In 130 nm CMOS technology, the resolution of this architecture is limited to about 50 ps. The use of more advanced architectures enabled the achievement of 18 ps-rms precision at the cost of increases in area, complexity and power consumption [11]. Delay-locked loops can be used in combination with linear interpolation to improve performance. Precision values of 1.35 ps-rms over a range of 25 ns have been achieved in a 64-channel device built in 65 nm CMOS technology [12]. It required full-custom optimization of digital cells and on-chip tuning of the delay taps and buffers. The large area and high power consumption make this solution nonideal for integration into monolithic detectors.

The Vernier TDC architecture improves precision beyond the constraints of the minimum delay unit by using two parallel delay lines with slightly different stage delays. The resolution is determined by the difference in delays between the two lines. However, a drawback of this approach is that the latency is not constant and increases with the duration of the measured time interval. A precision of 8.5 ps-rms was reported in 130 nm CMOS technology [13]. Linear Verniers use open-ended delay lines and are demanding in terms of area when a large FSR is needed. To extend the range with limited area, cyclic Vernier structures use delay lines closed in a loop. The latency of a linear Vernier does not introduce dead time because multiple measurements can be injected in the same line. However, the latency introduces dead time in cyclic structures, limiting the measurement rate.

TAC-based TDCs convert time intervals into an analog voltage that is then digitized by an ADC. They are particularly relevant in applications where picosecond precision is required along with high-speed operation and minimal latency. Moreover, time resolution does not depend on the intrinsic gate delay, and the dynamic range is not proportional to the area. Therefore, this architecture is well suited for any submicron technology node. A conventional TAC operates by charging a capacitor with a constant current during the time interval of interest and measuring the resulting voltage across it. Calibration is required to account for component variations, temperature, and nonlinearity in the analog circuit. The measurement rate is primarily constrained by the settling time of the TAC and the ADC sampling rate. The state-of-the-art implementation achieves 1.9 ps-rms over a 12.5 ns range but relies on BiCMOS technology, averaging of multiple samples, and a 70 mW power budget, precluding its use in high-density monolithic systems [9].

## 2. Materials and Methods

### 2.1. The Proposed TDC Architecture

The TDC architecture presented in this paper is based on the patent EP3591477B1 [14]. The objective is to obtain the time of arrival of an event $t_{0}$ by sampling two periodic signals with equal frequency and an offset in phase. Using a periodic signal v(t)=f(θ(t))=f(ωt+ϕ) with constant angular frequency $\omega =\frac{2\pi }{T}$, period *T*, and phase ϕ, the time corresponding to a certain value of *v* within a period can be determined using the inverse function $t=\frac{T}{2\pi }\left[f^{-1}\left(v\right)-\phi \right]$. While the choice of *f* is arbitrary, the feasibility of the system implementation should also be considered. In the simplified case of a noiseless system, the time resolution is strictly related to the sampled signal slope, $S=\frac{df}{dt}$, and the resolution of the ADC, Δv. The smallest possible time bin is then equal to $\Delta v{\left(\frac{df}{dt}\right)}^{-1}$. In a noisy system with a voltage noise $\sigma_{v}$, the precision is degraded by the jitter $\sigma_{t}=\sigma_{v}{\left(\frac{df}{dt}\right)}^{-1}$. Therefore, a constant $\frac{df}{dt}$ realizes a system with constant precision across the full period, making a sawtooth signal a straightforward choice for *f*. However, the use of such a function is impractical, as it would require a zero fall time in the abrupt discontinuity. Consequently, we require a continuous periodic function. Most of these, such as trigonometric functions, are not bijective, preventing the definition of a unique inverse function. In practice, this implies that sampling a periodic signal leads to ambiguities in time reconstruction, as the same amplitude value corresponds to multiple time bins within a single period. To resolve this, the architecture uses dual-phase periodic continuous signals $y\left(t\right)=f\left(\omega t+\phi_{y}\right)$ and $x\left(t\right)=f\left(\omega t+\phi_{x}\right)$ with the same frequency $freq=\frac{\omega }{2\pi }$ and a constant phase difference $|\phi_{y}-\phi_{x}|$. These signals are referred to as the reference signals throughout this work. The time information can be decoded with a linear calibration curve $t=a\;\cdot \;g^{-1}\left[y\left(t\right),x\left(t\right)\right]$ where $a=\frac{T}{2\pi }$ and $g^{-1}$ is the inverse function of the composite function g[y(t),x(t)].

**Figure 1:**
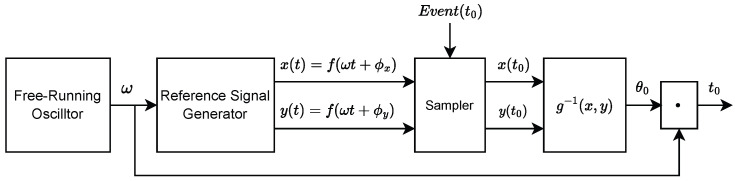
Block diagram of the proposed dual-phase TAC.

Figure 1 illustrates the block diagram of the architecture. A free-running oscillator generates a reference angular frequency ω, and a reference signal generator defines the two periodic signals x(t) and y(t) with period $T=\frac{2\pi }{\omega }$, which are sampled at the arrival of an event at $t_{0}$. Finally, the sampled signals $x(t_{0})$ and $y(t_{0})$ are used to reconstruct the time value $t_{0}$, as described above. The oscillator frequency value is measured and provided to the last calibration block. Multiple samplers can be used to measure multiple time intervals in parallel with a unique pair of reference signals.

As will be illustrated in Section 3.1, if the angular velocity ω is not constant within a period, the system can be calibrated with a look-up table. In this work, we used two sinusoid-like signals with a phase shift of $\frac{\pi }{2}$. With this choice, we can use $g^{-1}\left(y,x\right)=\mathrm{atan}2(y,x)$ to reconstruct the angle θ over the full range [0,2π], which corresponds to one complete period. Counting the number of reference signals periods occurring between the two events provides a coarse time measurement and extends the dynamic range of the system to multiple oscillation periods.

The time interval $\Delta t_{1\to 0}$ between two events occurring at $t_{0}$ and $t_{1}$ is therefore calculated as the sum of the coarse and fine time components.

(1) $$\Delta t_{1\to 0}=\Delta t_{1\to 0,\;\mathrm{fine}}+\Delta t_{1\to 0,\;\mathrm{coarse}}$$

(2) $$\Delta t_{1\to 0,\;\mathrm{fine}}=\frac{T}{2\pi }\left(\mathrm{atan}2\left(\frac{y_{1}}{x_{1}}\;\right)-\mathrm{atan}2\left(\frac{y_{0}}{x_{0}}\;\right)\right)$$

(3) $$\Delta t_{1\to 0,\;\mathrm{coarse}}=N_{c}T$$

where $N_{c}$ is the count of elapsed periods and $\left(x_{i},y_{i}\right)$ are the sampled values of the two reference signals at time $t_{i}$.

The proposed solution comes with advantages in terms of precision, high event-rate and scalability. The single-shot precision depends only on noise and ADC resolution; it is not limited by the technology node. The time-to-analog conversion has zero latency and ideally zero dead time. It does not depend on the time interval, and a full flash conversion could be achieved in combination with a flash ADC. The inherent latency introduced by other digitizer architectures, such as Successive-Approximation-Register ADC (SAR), can be compensated for with multi-event analog buffers. This architecture is scalable to a large number of channels, as several sampling channels can share the same oscillator and ADCs. It also allows for limited area, as well as for single calibration for multiple channels. Moreover, the power consumption is dominated by the signal generator, with little dependency on the number of channels, a feature that increases scalability. The free-running approach as a signal generator allows for easier implementation and reduced area as it does not require a phase-locking circuit. As a drawback, to achieve the best performance, it requires calibration based on a look-up table.

### 2.2. The Demonstrator ASIC

**Figure 2:**
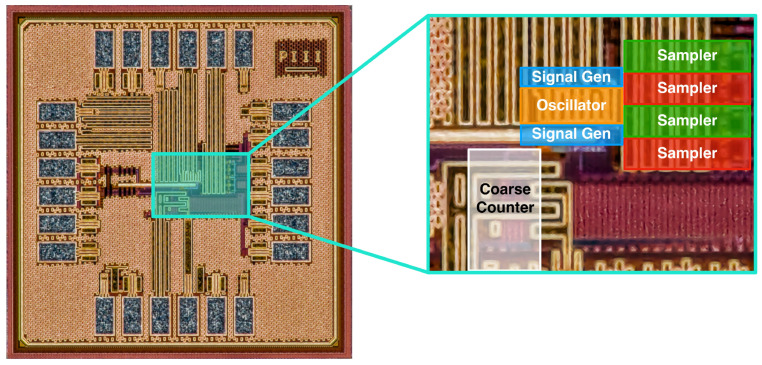
Microphotograph of the proof-of-concept ASIC. The area where the architecture is implemented is magnified, and the main building blocks are highlighted with the same color code used in Figure 3.

**Figure 3:**
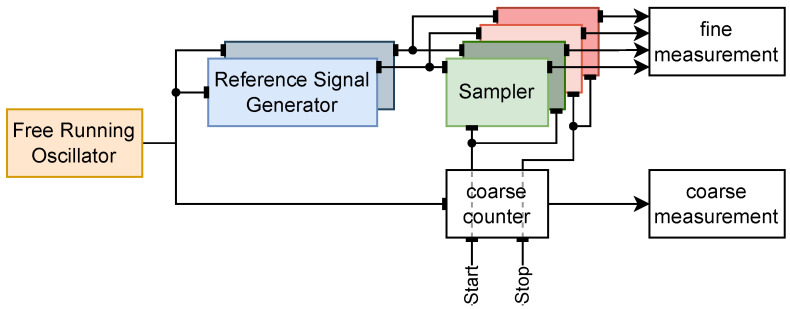
Block diagram of the proof-of-concept ASIC.

**Figure 4:**
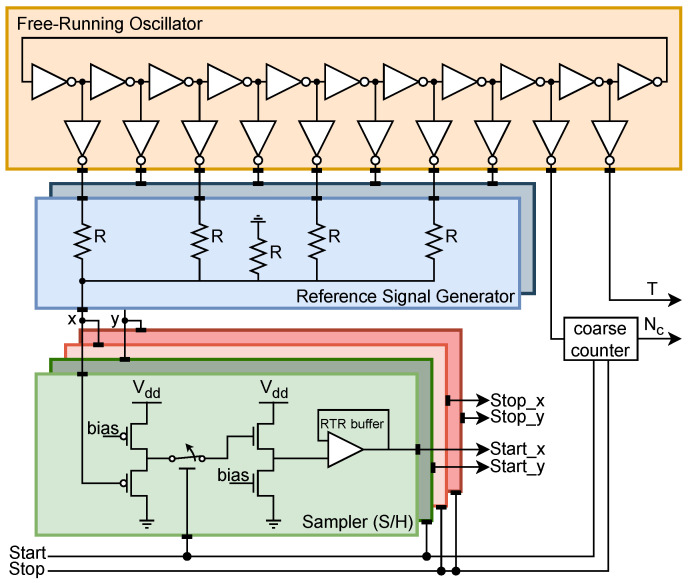
Schematic diagram of the proof-of-concept ASIC.

The proof-of-concept ASIC (Figure 2) implements a start channel and a stop channel to allow the measurement of intervals between two events. The block diagram and the schematic of the chip are shown in Figure 3 and Figure 4, respectively. It uses an 11-stage single-ended CMOS free-running oscillator with a frequency ranging from 0.89 GHz to 1.61 GHz for power-supply voltages from 1.2 V to 1.6 V, respectively. The nodes of the oscillator are buffered to a resistive interpolator circuit that generates the dual-phase reference signals. Each branch of the interpolator is composed of four resistors $R_{0,1,2,3}$, which serve as weights, alongside a reference resistor *R*; together, they form an analog adder. The resistors are implemented in high-resistive poly-silicon to keep the layout compact. The interpolation produces signals with steep steps between peaks caused by the flip of a ring oscillator stage. The parasitic capacitance at the nodes smooths the curves, generating sinusoidal-like signals. Each event channel has two independent sampler circuits composed of a Sample and Hold (S/H) and a rail-to-rail (RTR) buffer. The RTR buffers are not optimized for settling time, since this prototype was intended to be a demonstrator for the working principle and not to probe the maximum event rate. In parallel with the TAC, a counter is used to track the number of oscillator revolutions gated between start and stop, with the result providing the coarse time measurement. The counter is implemented with nine-bit Linear Feedback Shift Registers (LFSRs) ensuring a range of 511 period counts and high-speed counting. In this work, we report the results for a maximum range of 25 ns. The coarse measurements are stored in a shift register and serialized to the output with the use of an external clock. One of the nodes of the ring oscillator is buffered to a pad to monitor the ring oscillator frequency.

**Figure 5:**
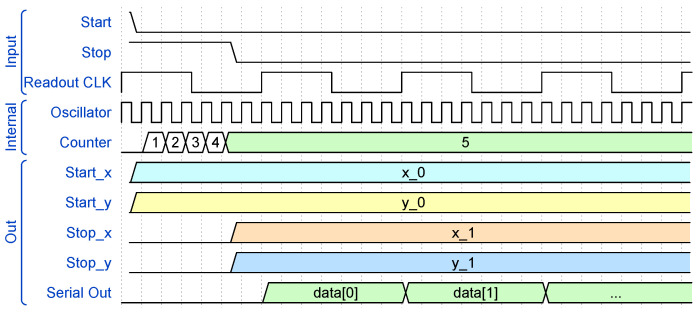
Simplified timing diagram of the ASIC operation.

Figure 5 illustrates a simplified timing diagram of the chip operation. At the falling edges of the start and stop signals, the two couples $\left(x_{0},y_{0}\right)$ and $\left(x_{1},y_{1}\right)$ are sampled and buffered and the LFSR counter is enabled between the two. On the first readout-clock tick after the stop falling edge, the counter value is serialized.

### 2.3. Single-Shot Precision Analysis

This section analyzes the primary sources of jitter affecting system precision, with the objective of deriving key design parameters for optimal implementation.

The measurement performed by the system in Figure 1 is affected by two sources of uncertainty: the one-period jitter $\sigma_{T}$ and the uncertainty in angle measurement $\sigma_{\theta }$. Using Equation (1), we can express the TDC precision $\sigma_{t}$ as follows:

(4) $$\sigma_{t}^{2}=\sigma_{t,\mathrm{fine}}^{2}+\sigma_{t,\mathrm{coarse}}^{2}$$

#### 2.3.1. Uncertainty on the Fine Measurement

$\sigma_{t,\mathrm{fine}}$ corresponds to the greatest value of jitter that the system can achieve.

We can derive it from Equation (2):

(5) $$\sigma_{t,\mathrm{fine}}^{2}=\frac{\theta^{2}}{4\pi^{2}}\sigma_{T}^{2}+\frac{T^{2}}{4\pi^{2}}\sigma_{\theta }^{2}\approx \frac{T^{2}}{4\pi^{2}}\sigma_{\theta }^{2}$$

since the second term dominates over the first one. The uncertainty $\sigma_{\theta }$ can be derived by examining the diagram in Figure 1. The phase θ is measured through the reference signals x(t) and y(t), which are generated with an interpolator. The output of the interpolator v(t) is described by

(6) $$v\left(t\right)=\sum w_{i}o_{i}\left(t\right)$$

where *i* is the index of the oscillator node and $w_{i}$ and $o_{i}\left(t\right)$ are the weight and the output of the *i*-node, respectively. The two reference signals use different weights $w_{i}$ to achieve the desired phase shift.

Let us consider the uncertainty associated with the nodes of the free-running oscillator $o_{i}$. In Hajimiri et al. [15], the jitter of such a system is separately well described for short and long intervals of observation. For short intervals, we can consider the noise sources of each node of the oscillator as not being correlated with the ones affecting the other nodes. In long intervals, the correlation between the noise sources of different nodes becomes dominant, mainly because of the low-frequency noise. Since $\sigma_{\theta }$ is the uncertainty of the measurement of an angle within a single oscillation period. we can consider only uncorrelated noise sources for each of the oscillator nodes $\sigma_{o_{i}}$ and derive the voltage noise $\sigma_{v}$ as follows:

(7) $$\sigma_{v}^{2}=\sum w_{i}^{2}\sigma_{o_{i}}^{2}+\sigma_{\mathrm{sampler}}^{2}$$

where we included the contribution of the S/H and ADC, both described by $\sigma_{\mathrm{sampler}}$.

The sampled values are then converted into time with the calibration function t=a·g(y,x), and in this work, we adopt g(y,x)=atan2(y,x). Propagating the uncertainty of the reference signals to the angle, we obtain

(8) $$\theta =\mathrm{atan}2\left(\frac{y_{1}}{x_{1}}\right)-\mathrm{atan}2\left(\frac{y_{0}}{x_{0}}\right)$$

To further simplify, we consider measurements of a multiple of the period, and therefore, $x=x_{0}=x_{1}$ and $y=y_{0}=y_{1}$

(9) $$\frac{\sigma_{\theta }^{2}}{2}={\left(\frac{x}{x^{2}+y^{2}}\right)}^{2}\sigma_{y}^{2}+{\left(\frac{y}{x^{2}+y^{2}}\right)}^{2}\sigma_{x}^{2}$$

$$\mathrm{where}\;\left\{\begin{array}{cc} \frac{\partial }{\partial x}\mathrm{atan}2\left(\frac{y}{x}\right) & =-\frac{y}{x^{2}+y^{2}},\\ \frac{\partial }{\partial y}\mathrm{atan}2\left(\frac{y}{x}\right) & =\frac{x}{x^{2}+y^{2}} \end{array}\right.$$

The two signals are generated by equivalent systems, therefore $\sigma_{y}=\sigma_{x}=\sigma_{v}$ and

(10) $$\sigma_{\theta }^{2}=\frac{2}{x^{2}+y^{2}}\sigma_{v}^{2}$$

(11) $$\sigma_{\theta }=\frac{\sqrt{2}}{r}\sigma_{v}$$

where $r=\sqrt{x^{2}+y^{2}}$. Finally, we can propagate the uncertainty again:

(12) $$\sigma_{t,\mathrm{fine}}=\frac{T}{2\pi }\sigma_{\theta }=\frac{T}{\sqrt{2}\pi r}\sigma_{v}$$

The jitter of the fine measurement is therefore proportional to the oscillator period and inversely proportional to *r*. This consideration does not take into account possible variation in the angular velocity within a period. As mentioned, we have calibrated the system with a look-up table to compensate for nonconstant velocity when transforming the angle values into time, as reported in Section 3.1.

#### 2.3.2. Uncertainty on the Coarse Measurement

The uncertainty $\sigma_{t,\mathrm{coarse}}$ can be derived from Equation (3)

(13) $$\sigma_{t,\mathrm{coarse}}^{2}=N_{c}^{2}\sigma_{T}^{2}$$

where $N_{c}$ is assumed free of uncertainty. To measure the oscillator period *T*, we average several oscillator cycles:

(14) $$T=\frac{\sum_{i=0}^{M}T_{i}}{M}$$

where *M* is the number of cycles averaged and $T_{i}$ is the period associated with *i*-cycle.

We can describe the period jitter as

(15) $$\sigma_{T}^{2}\approx \frac{\sigma_{T\_uncorr}^{2}}{M}+\sigma_{T\_corr}^{2}$$

where $\sigma_{T\_uncorr}$ and $\sigma_{T\_corr}$ are the one-period jitter caused by uncorrelated and correlated noise sources across the oscillator nodes [15]. While the uncorrelated noise can be minimized with averaging, the period jitter $\sigma_{T}$ is still limited by the correlated noise. Therefore, to minimize the uncertainty on the period measurement, particular effort was devoted to optimizing the layout and minimizing correlated noise, mainly that originating from power supply and parasitic coupling between nodes.

#### 2.3.3. Precision for Short and Long Ranges

Finally, we can simplify Equation (4), depending on the value of $N_{c}$, comparing Equations (5) and (13):

(16a) $$\begin{array}{ccccc} \sigma_{t} & \approx \sigma_{t,\mathrm{fine}\;} & N_{c}\ll \frac{T}{\sigma_{T}}\;\frac{\sigma_{\theta }}{2\pi } & \; & \mathrm{Short}\;\mathrm{Range} \end{array}$$

(16b) $$\begin{array}{ccccc} \;\sigma_{t} & \approx \sigma_{t,\mathrm{coarse}\;} & N_{c}\gg \frac{T}{\sigma_{T}}\;\frac{\sigma_{\theta }}{2\pi } & \; & \mathrm{Long}\;\mathrm{Range} \end{array}$$

The precision is constant for short intervals and can be approximated to $\sigma_{t,\mathrm{fine}}$, while it is proportional to the number of elapsed periods $N_{c}$ for longer intervals, since $\sigma_{t,\mathrm{coarse}}\propto N_{c}$.

### 2.4. Measurement Setup

**Figure 6:**
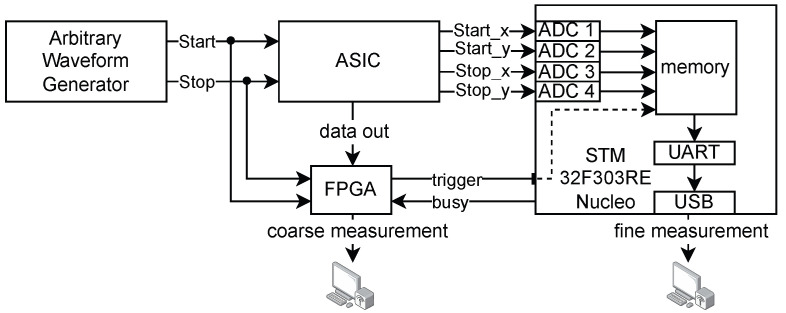
Block diagram of the measurement setup.

The experimental setup used to obtain the measurements presented in this paper is illustrated in Figure 6. An arbitrary waveform generator (Keysight M8195A [16]) provides start and stop signals with a measured jitter below 600fs. A copy of the two signals trigger the readout of a custom FPGA-based DAQ, which handles the digital readout of the coarse counter. The DAQ forwards the trigger to the external ADCs of a STM32F303RE Nucleo [17] that operate the voltage-to-digital conversion of the four samples of the reference signals from the chip. The ADCs are operated in continuous conversion mode, and the samples are stored at the arrival of the trigger and sent to a PC via a USB-UART interface. To ensure synchronization during the two phases of conversion and transmission, the STM board sends a busy signal to prevent the FPGA from accepting new measurements while UART transmission is occurring, avoiding conflicts in data acquisition. The ADCs operate at 12-bit resolution with an LSB of 0.8 mV on a full-scale range of 3.3 V, while the output voltage of the TAC has a maximum range of 1.1 V. The ADC conversion time is 250 ns.

## 3. Results and Discussion

### 3.1. System Calibration

**Figure 7:**
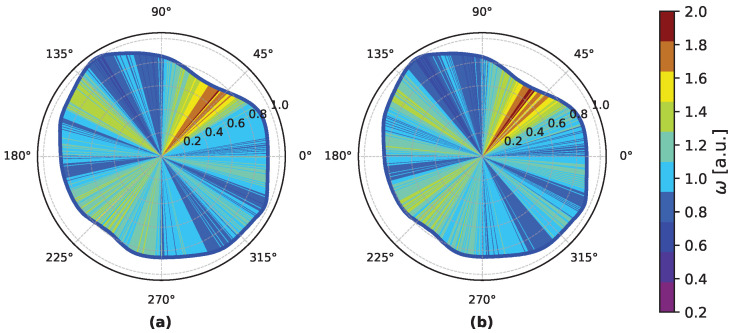
Polar plot of the reference signals measured in the start (**a**) and stop (**b**) channels. The full 2π angle is divided in $N_{bins}$ equally spaced in time, and each of them is colored depending on its angular velocity ω.

To calibrate the system, the amplitudes pairs $\left(x,y\right)$ are converted to polar coordinates in the range (0,2π], as illustrated in Figure 7. A single revolution of the curve corresponds to one period of the oscillator. The choice of the origin θ=0 is arbitrary, and it is chosen such that it corresponds to the sensitive edge of the coarse counter. In the polar coordinate system, the time interval between the two events is represented by the angle between the position vectors of the two samples.

In the case of nonideal sinusoidal signals, the angular velocity must be calibrated to ensure time uniformity for each angular bin. This means that different angle bins cover different $\Delta \theta_{i}$ but represent equal time intervals Δt; therefore, each bin has a different angular velocity $\omega_{i}=\frac{\Delta \theta_{i}}{\Delta t}$. We collected *S* samples of the reference signals x(t) and y(t) at random time intervals and calculated the corresponding angle $\theta =\mathrm{atan}2(\frac{y}{x})$. The full angle of 2π rad was then divided into $N_{bins}$ nonuniform angular bins, each containing the same number of samples $\frac{S}{N_{\mathrm{bins}}}$, with *S* an integer multiple of $N_{\mathrm{bins}}$. This calibrates the bins into equal time windows, which defines the system resolution as $\mathrm{LSB}=T/N_{bins}$. While calibrating each of the two channels, the other one was left open to prevent any interchannel interference. In this polar coordinate system, the module of the position vector $r=\sqrt{x^{2}+y^{2}}$ does not include relevant information for the time conversion and can be ignored. However, Equation (11) describes the systematic error, depending on *r*. Therefore, it is important to have a sufficient number of samples per bin to minimize the systematic calibration error. The colors of Figure 7 illustrate the ratio between the calibrated angular bin width and the ideal one $\frac{2\pi }{N_{bins}}$. This ratio is proportional to the angular velocity of the system in each bin. In the same figure, *r* is normalized to its maximum.

**Figure 8:**
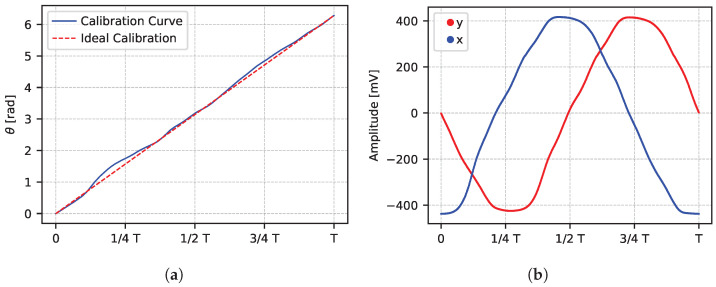
(**a**): The calibration curve (blue line) compared to the ideal calibration (dashed red line). (**b**): The two reference signals (x,y) reconstructed after calibration.

Figure 8a shows the calibration curve in comparison with the ideal calibration. The calibration curve is used as a look-up table to convert angle information into time. The reconstructed reference signals are shown in Figure 8b.

The period *T* can be calibrated either by using the dedicated frequency-monitor pad or by measuring a defined time interval and inverting Equation (1). The latter method was used in this work. The following results were achieved with calibration in post-processing dividing the period T=(616.9±0.1) ps in $N_{bins}=650$, and resulting in an LSB of 0.95 ps.

### 3.2. Single-Shot Precision

**Figure 9:**
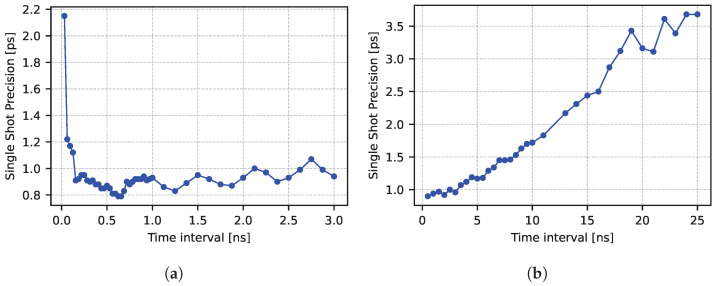
Single-shot precision for short (**a**) and long (**b**) time intervals.

The TDC was tested with different Δt to characterize the performance at different ranges. Figure 9a shows the results for time intervals up to five oscillator periods. Time intervals below 0.2 ns suffer from increased jitter due to interference between start and stop channels, which can cause a perturbation of the reference signals. For intervals between 0.2 ns and 3 ns, the TDC shows an approximately constant single-shot precision, as expected in short ranges, with an average value of about 0.9 ps-rms, and a minimum of 0.79 ps-rms. The precision exhibits a periodic variation of about 0.1 ps, with a minimum occurring at values of Δt that are integer multiples of *T*. We expect systematic errors affecting the fine time to cancel out every *T*, since the interval measurement relies on the difference of values that share the same error (Equation (2)). Examples of these possible error sources include digitization and calibration error. A similar effect can be caused by a low-frequency noise that modulates the amplitude of the reference signals. If this modulation frequency is significantly lower than the oscillator one, the resulting degradation in precision will be compensated for if the time interval is an integer multiple of the period and will show a maximum at odd half-multiples of *T* (i.e., 1/2T, 3/2T, …). We reproduced both effects using Monte Carlo simulations, with results that confirmed the periodic pattern of the precision.

Figure 9b illustrates the precision for longer intervals. The system precision shows an approximately linear behavior with respect to the number of cycles $N_{c}$, in agreement with Equation (16b), with the largest tested interval of 25 ns achieving 3.7 ps-rms.

**Figure 10:**
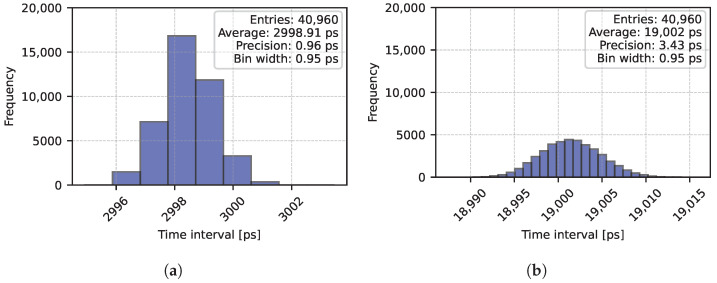
Sample histograms for time intervals of 3 ns (**a**) and 19 ns (**b**).

As an example, we report in Figure 10 the histograms of time intervals of 3 ns and 19 ns, achieving a precision of 0.96 ps-rms and 3.43 ps-rms, respectively, with a Gaussian distribution.

### 3.3. Linearity

**Figure 11:**
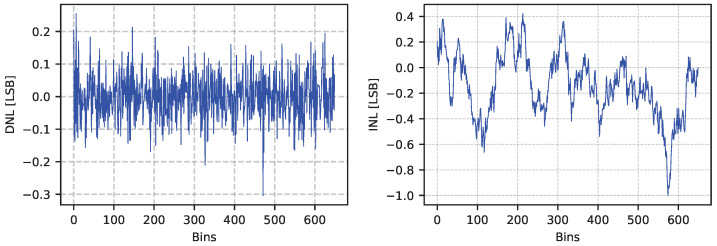
Measured DNL and INL.

The DNL and and integral-nonlinearity (INL) were computed for both start and stop sampling channels. They were calculated after calibration of the angular velocity with a set of independent samples for each channel at random times, accumulating on average 2000 samples per bin. The DNL was estimated as the difference in number of events between consecutive bins, while the INL was estimated as the cumulative sum of the DNL values within one period. Due to the periodic nature of the proposed TDC architecture, we report the peak-to-peak (p–p) values of DNL and INL as a worst-case estimate across the entire period. The plots of DNL and INL within one oscillator period are reported in Figure 11, with $DNL_{p\text{-}p}=0.56\;\mathrm{LSB}\;$ and $INL_{p\text{-}p}=1.43\;\mathrm{LSB}$.

### 3.4. Temperature Stability

**Figure 12:**
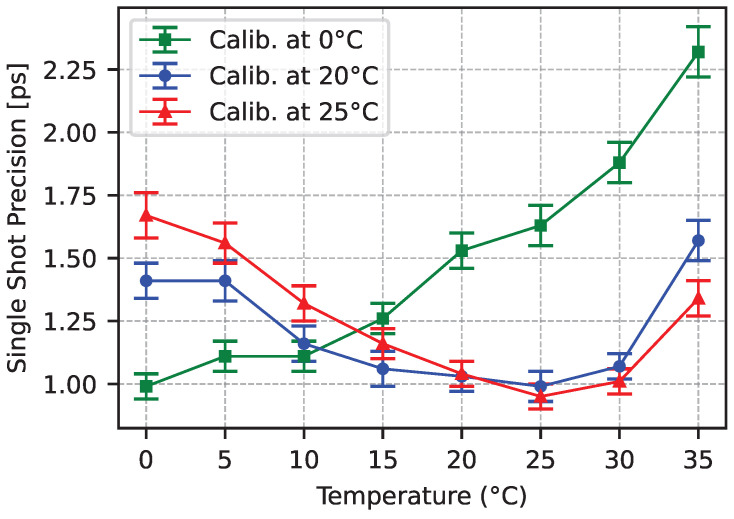
Single-shot precision for Δt=718 ps at various temperatures. For each of the three datasets, the calibration was performed at a the temperature reported in the legend. A performance plateau of approximately 5 °C is observed around the calibration point.

We have characterized the system at different temperatures ranging from 0 °C to 35 °C, measuring a time interval of 718 ps. We first calibrated the angular velocity at three different temperatures: 0 °C, 20 °C, and 25 °C. Then, we post-processed the datasets using the three calibration curves while adjusting only the LSB for each of the temperatures. The results shown in Figure 12 demonstrate that the same precision of approximately 1 ps can be achieved at any of the tested temperatures after calibration of both the angular velocity and the LSB. Calibrating only the LSB allows for stable operation at ±5 °C with a precision below 2 ps-rms for a wide range of temperatures.

### 3.5. Count Rate and ADC Resolution

While the ASIC design was not optimized for conversion speed, we can estimate the maximum possible event rate for this architecture. The coarse measurement is immediate. In the case of the fine measurement, the conversion time depends on the sampler settling time and on the ADC. The current prototype is limited by the RTR buffer to 22 Mcps. An integrated chip including both TAC and ADC can therefore achieve conversion times on the order of a few nanoseconds, as the conversion rate will depend mainly on the ADC conversion time. Considering 8 ns digital conversion time [9] and a single sample, we can predict an upper limit for the count rate on the order of 100 Mcps.

**Figure 13:**
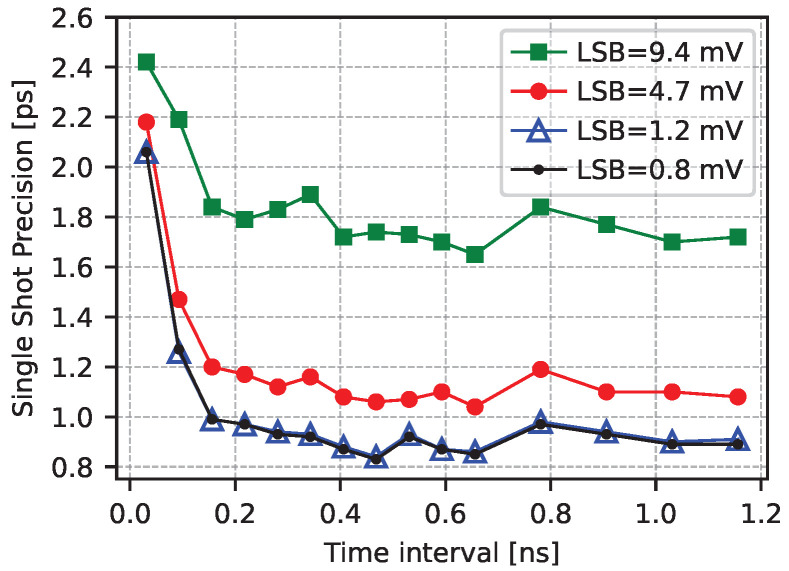
Single-shot precision after resampling for different resolutions. LSBs of 1.17 mV, 4.69 mV, and 9.38 mV correspond to 10-, 8-, and 7-bit ADCs with a 1.2 V FSR, respectively. The precision with an LSB of 0.8 mV, as in previous studies, is reported in the plot for comparison.

Finally, to assess the impact of the ADC resolution on the single-shot precision, we analyzed the performance of the system in post-processing, resampling the ADC values for different LSB values, as shown in Figure 13. An LSB of 1.2 mV, equivalent to a 10-bit ADC with an FSR of 1.2 V, does not degrade the performance of the system compared to the measurement presented in Figure 9a (LSB of 0.8 mV). The ADC resolution can be further reduced to eight or seven bits, with slight degradation in the precision of the TDC.

## 4. Conclusions

This paper presents a novel TDC architecture based on the analog sampling of dual-phase signals from a free-running oscillator. The architecture is intended for systems requiring a large number of channels and count rates on the scale of tens of Mcps.

The results obtained with a proof-of-concept ASIC demonstrate an average precision of about 0.9 ps-rms for time intervals up to 3 ns and a precision below 3.7 ps-rms for time intervals up to 25 ns. The architecture allows for scaling-up to multiple channels with a single reference generator that occupies a very small area and utilizes little power.

This design is compared to other TDCs available in the literature in Table 1. To our knowledge, the proof-of-concept ASIC presented here demonstrates the best precision for a fully-integrated TAC architecture and the target FSR in a compact area, enabling high-channel-density integrated implementations. The architecture does not require any averaging of multiple measurements, and the measurement latency depends only on the sampler settling time and the choice of ADC architecture.

## 5. Patents

The work is based on the patent EP3591477B1 “Device and method for measuring the relative time of arrival of signals”.

## Figures and Tables

**Table 1:** Comparison of different works on TDCs.

	This Work		[9]		[7]	[18]	[8]	[19]	[12]	
Application	**TCSPC/HEP**		TCSPC		PLL	Imaging	DPLL	Medical Imaging	HEP	
Architecture	**TAC + counter**		TAC		TAC + ADC	DEM Vernier ^a^	2-D spiral Vernier	TDC VGRO ^b^	DLL	
Process [nm]	**130 CMOS**		350 BiCMOS		65 CMOS	28 CMOS	45 CMOS	130 CMOS	65 CMOS	
Range [ns]	3	25	12.5	100	±0.41	17.4	±0.32	9	204,000	
Resolution [ps]	**0.95**		0.782	6.1	0.8	8.5	1.25	7.3	3	12
Bits	**23** ^3^	**26** ^c^	14		10	11	8	7	26	
Precision min [ps-rms]	**0.79**		1.9	-	1.1	7.5	0.35	8.4	1.35	3.65
DNL max [LSB]	**0.56**		0.018	-	1	0.23	0.25	3.2	-	
DNL RMS [LSB]	**0.07**		-	0.13	0.08	-	-	0.8	0.82	
INL max [LSB]	**1.43**		0.025	-	3.1	2.3	0.34	3.5	0.44	
INL RMS [LSB]	**0.25**		-		-	-	-	1.2	0.14	
Count Rate [Mcps]	**22**		12.3		50	15	80	2.4	-	
Power per channel [mW]	**4.1** ^d^		70		2.9	0.2	0.69	1.2	20	13
Area [${\mathrm{mm}}^{2}$]	**0.0014** ^e^		0.2 ^f^		0.02	0.006 ^g^	0.04 ^h^	0.03	1.14 ^i^	

Notes: ^a^ DEM: Dynamic Element Matching. ^b^ VGRO: Vernier Gated-Ring Oscillator. ^c^ 10-bit ADC for each reference signal, as in Figure 13, plus 3 or 6 bits for the coarse counter. ^d^ Includes one sampling channel and coarse counter. In addition, 4 mW are needed for the reference signal generators. ^e^ Includes oscillator, reference generator, start and stop samplers, and 6-bit coarse counter. ^f^ Additional DAC area of 0.13 mm^2^ can be shared with multiple channels. ^g^ Additional DEM with an area of 0.12 mm^2^ (65 nm). ^h^ Does not include auxiliary circuits. ^i^ Estimated area of a single channel plus DLL. Full ASIC area 20.25 mm^2^.

## Data Availability

The raw data supporting the conclusions of this article will be made available by the authors on request.
